# Polphylipoprotein-induced autophagy mechanism with high performance in photodynamic therapy

**DOI:** 10.1038/s42003-023-05598-0

**Published:** 2023-11-28

**Authors:** Atsushi Taninaka, Hiromi Kurokawa, Mayuka Kamiyanagi, Takahiro Ochiai, Yusuke Arashida, Osamu Takeuchi, Hirofumi Matsui, Hidemi Shigekawa

**Affiliations:** 1 Faculty of Pure and Applied Sciences, University of Tsukuba, 1-1-1 Tennodai, Tsukuba, Ibaraki 305-8573 Japan; 2 TAKANO Co. LTD. Miyada-mura, Kamiina-gun, Nagano, 399-4301 Japan; 3 Fuculty of Medicine, University of Tsukuba, 1-1-1 Tennodai, Tsukuba, Ibaraki 305-8575 Japan

**Keywords:** Cancer therapy, Autophagy

## Abstract

Polphylipoprotein (PLP) is a recently developed nanoparticle with high biocompatibility and tumor selectivity, and which has demonstrated unprecedentedly high performance photosensitizer in photodynamic therapy (PDT) and photodynamic diagnosis. On the basis of these discoveries, PLP is anticipated to have a very high potential for PDT. However, the mechanism by which PLP kills cancer cells effectively has not been sufficiently clarified. To comprehensively understand the PLP-induced PDT processes, we conduct multifaceted experiments using both normal cells and cancer cells originating from the same sources, namely, RGM1, a rat gastric epithelial cell line, and RGK1, a rat gastric mucosa-derived cancer-like mutant. We reveal that PLP enables highly effective cancer treatment through PDT by employing a unique mechanism that utilizes the process of autophagy. The dynamics of PLP-accumulated phagosomes immediately after light irradiation are found to be completely different between normal cells and cancer cells, and it becomes clear that this difference results in the manifestation of the characteristic effect of PDT when using PLP. Since PLP is originally developed as a drug delivery agent, this study also suggests the potential for intracellular drug delivery processes through PLP-induced autophagy.

## Abstract (web summary)

A photodynamic therapy with polphylipoprotein (PLP) was conducted using both normal cells and cancer cells originating from the same sources. PLP enables highly effective cancer treatment by employing a unique mechanism of autophagy.

## Introduction

Photodynamic therapy (PDT) treats diseases by dosing photosensitizers into a body and irradiating them with light to generate reactive oxygen species (ROS)^1–3^. It is noninvasive with few adverse effects and exhibits a high therapeutic effect on lesions that are difficult to treat by surgical techniques. It is used not only for cancer therapy but also for conditions such as age-related macular degeneration and skin diseases^4–6^. Recently, antimicrobial PDT has been developed as a method to efficiently inactivate pathogens without inducing resistant bacteria, and it has been applied to treat periodontal disease^7–10^. It also has been reported that PDT has an abscopal effect: the shrinkage of untreated tumors was concurrently observed with the shrinkage of tumors upon local treatment^11–13^.

**Fig. 1:**
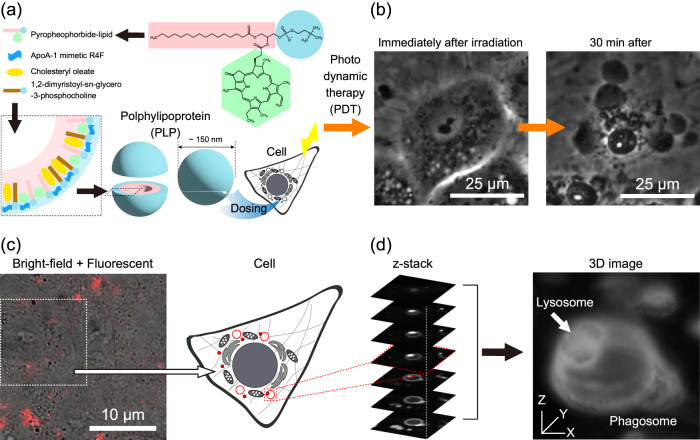
Schematic diagram of PDT with PLP. **a** Structure of polphylipoprotein (PLP). **b** Phase-contrast images of RGK1 immediately after and 30 min after irradiation with light of 655 nm for 1 min. After 30 min, blebs were formed, and the cells became necrotic. **c** Overlap image of bright-field and fluorescence images of a PLP-administered RGK1 sample. The fluorescent images are pseudocolored in red. **d** Series of z-stack fluorescence images of a phagosome after PLP administration (left) and three-dimensional (3D) image formed using the z-stack images (right). The structure of phagolysosome, the binding structure between phagosome and lysosome, is clearly visible, where the lysosome is indicated by the white arrow.

The observed effect of PDT mainly depends on the chemical properties of the photosensitizer. Therefore, various photosensitizers suitable for treatment with PDT, for example, porfimer sodium and talaporfin sodium, have been developed^14^. Polphylipoprotein (PLP) is a recently developed nanoparticle with high biocompatibility and tumor selectivity^11,15,16^, which has demonstrated unprecedentedly high performance as a photosensitizer in PDT and photodynamic diagnosis (PDD). Figure 1a shows the structure of PLP. It is a porphyrin-base nanoparticle consisting of imitated lipoprotein formed by lipids and proteins, with a core-shell structure, hydrophilic functional groups, and hydrophilic molecules on the outside hydrophobic functional groups and hydrophobic molecules on the inside. Pyropheophorbide-lipid, a component of PLP, has phospholipids and a chlorin ring and forms a spherical structure with the hydrophobic chlorin ring arranged inside. Although PLP was originally developed as a drug delivery agent^15,16^, it has been investigated as a photosensitizer with high performance in PDT and PDD.

PDT using PLP, similarly to conventional PDT, generates ROS by dosing cells with PLP followed by light irradiation, which is considered to destroy lesions and exert a therapeutic effect (Fig. 1b). However, this mechanism does not provide sufficient understanding of why PLP enables the highly effective treatment of cancer cells in PDT. Recently, it has been revealed that PLPs accumulate on the phagosome membrane in the cells of RGK1, a rat gastric mucosa-derived cancer-like mutant^17^.

Phagosomes are vesicles in which parts of cytoplasm and foreign substances are surrounded by a phospholipid bilayer membrane called an isolation membrane and act in the process of autophagy^18,19^. Autophagy, the function of cells to degrade their unnecessary components, has a mechanism that begins with the generation of phagosomes. When PLPs are taken into a cell, they may be considered as targets to be degraded and sequestered by phagosomes^17^. If phagosomes accumulate PLPs, according to the autophagy mechanism, the PLP-accumulated phagosomes may bind to lysosomes, forming phagolysosomes, which normally degrade the taken substances using hydrolytic enzymes in the phagolysosomes. Figure 1c shows an overlap image of bright-field and fluorescence images of a PLP-dosed RGK1 sample. Figure 1d shows a series of z-stack fluorescence images of the phagosomes shown in Fig. 1c obtained by a confocal super-resolution microscope (left) in this study and a three-dimensional (3D) image produced from the z-stack images (right). As expected, the phagosome-lysosome combined structure is clearly visible.

If PDT is performed after phagolysosomes with accumulated PLPs are produced in a cell, the ROS generated by light irradiation destroys the membrane of the phagosomes, then, foreign substances the phagolysosomes, such as hydrolytic enzymes and ROS, diffuse into the cell, destroying organelles^17^. By these mechanisms, the cell undergoes necrosis, as shown in Fig. 1b^17,20^. This process convincingly explains why PLP demonstrates higher efficacy than other photosensitizers in PDT. However, since phagosomes are produced in all types of cells, such as cancer cells and normal cells, this mechanism of necrosis alone cannot explain the selective tumor treatment by PDT with PLP, which is highly effective only for cancer cells. Since PLP is a promising photosensitizer, clarification of its effective role in PDT is strongly desired.

To respond to this urgent requirement, we conducted multifaceted experiments using both normal cells and cancer cells originating from the same sources, namely, RGM1^21^, a rat gastric epithelial cell line, and RGK1^21^, a rat gastric mucosa-derived cancer-like mutant. The experiment comparing the effects of PDT using normal cells and cancer cells is a first-of-its-kind attempt. We revealed that PLP has a previously unreported mechanism that enhances the effectiveness of cancer treatment by inducing and utilizing autophagy. PLP-induced phagosome generation and its reaction processes upon light irradiation in PDT show entirely different behaviors in RGK1 and RGM1, explaining the mechanism of PDT by PLP with highly selective efficacy on cancer cells. Since PLP was originally developed as a drug delivery agent, this study also suggests the potential for intracellular drug delivery processes through PLP-induced autophagy.

## Results

### Sample preparation

RGM1 and RGK1 samples were prepared in *Φ* = 60 mm culture dishes with 30–50% cell area occupancy two days after passage. Details of the culture method are described in Methods. PLP was added to samples to a final concentration of 19 μM, and after allowing the samples to stand in an incubator for 24 h, they were washed with a new medium to remove the PLPs not taken up into the cells. The samples were placed in a top-stage incubator on an IX83 optical microscope system (Olympus Corp.), and experiments were carried out. For PDT measurements, samples were irradiated with light with wavelengths from 635 to 675 nm through a bandpass filter.

### Effect of PLP-PDT at the cell level

**Fig. 2:**
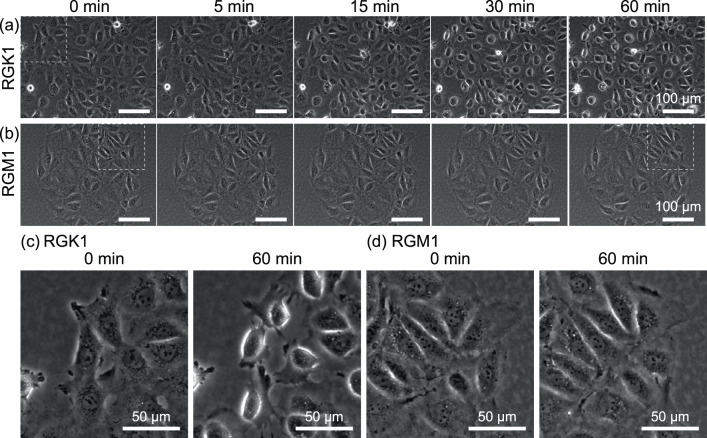
Phase-contrast images. **a** RGK1 and **b** RGM1 after irradiation of 28.7 mW cm^−2^ light with a central wavelength of 655 nm for 1 min. **c**, **d** Enlarged images of the squared areas in **a** and **b**, respectively. As evident in the enlarged images, from 0 to 5 min, the cells maintain the same shape. However, after that, in RGK1, the shape becomes rounded, indicating that RGK1 has sustained damage.

First, we observed the selective effects of PLP-induced PDT at the cell level. Figure 2 shows phase contrast images of (a) RGK1 and (b) RGM1 samples, which were irradiated with light of 28.7 mW cm^-2^ for 1 min. As shown in Fig. 2a, there was no deformation of the RGK1 at 5 min after the irradiation, but shrinkage was observed at 15 min. Thereafter, the cells had become rounded and were lifted off the bottom of the dish at 60 min (Fig. 2c). In contrast, as shown in Fig. 2b, there was no deformation of the RGM1 even at 60 min after the irradiation, and all cells migrated as usual (Fig. 2d). These results indicate that although the RGK1 cells were damaged, the RGM1 cells were undamaged by the PDT process.

**Fig. 3:**
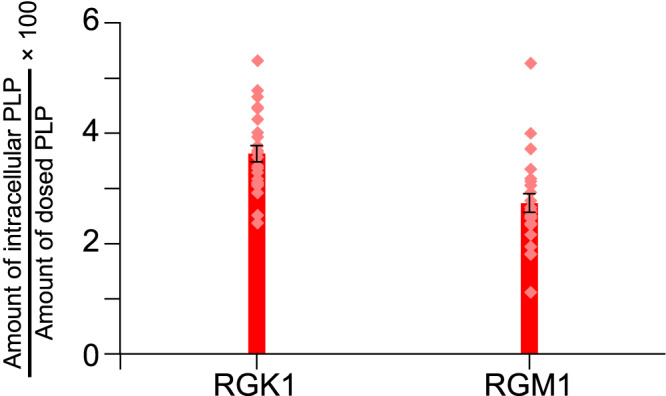
Ratio of PLP taken into cells to the dose for RGK1 and RGM1. Each amount of PLP was determined by the fluorescence intensity of PLP (*N* = 24). The rates are 3.6% for RGK1 and 2.7% for RGM1.

Since singlet oxygens (^1^O_2_), which are generated from photosensitizers by the PDT process, are transformed to other types of ROS and are considered to damage several cytoplasmic organelles^2,14,17,20,22,23^, the amount of photosensitizer s introduced into the cells may influence the effect of PDT observed for cancer and normal cells^17,20^. In fact, PDT with talaporfin sodium uses the difference in its amount taken up in cancer and normal cells. Namely, the PDT treatment is performed when the difference in the amounts of talaporfin sodium in cancer and normal cells is at its maximum^20^. Therefore, the same amounts of PLP were dosed into the RGK1 and RGM1 samples shown in Fig. 2. Namely, the incubation time after dosing PLP was set to 24 h, longer than the 3 h at which the difference in the amounts taken up is maximum. Figure 3 shows the ratios of intracellular PLP dosed into RGK1 and RGM1, determined from the fluorescence intensity of PLP (See Methods for detail). They were estimated to be 3.6% and 2.7%, respectively, and no significant difference in the observed PDT effect was obtained.

The difference in the effects of PDT with PLP on cancer cells and normal cells shown in Fig. 2 was confirmed at the cellular level. We next investigated the cause of this difference.

### PLP distributions in RGK1 and RGM1

**Fig. 4:**
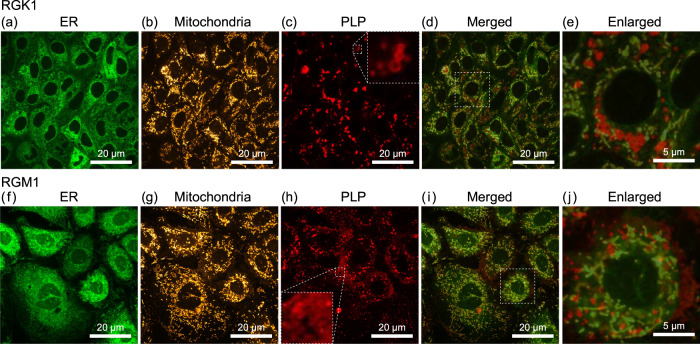
Super resolution fluorescence images. **a**, **f** Endoplasmic reticulum (ER) fluorescence images, **b**, **g** mitochondrial fluorescence images, **c**, **h** PLP fluorescence images, **d**, **i** merged ER, mitochondrial, and PLP images, and **e**, **j** enlarged images of the squares in **d** and **i** for the RGK1 and RGM1 samples, respectively. Red ring-shaped structures are observed in the insets of **c** and **h** and in **e** and **j**. ER-Tracker™ Green (Invitrogen™) and MitoTracker™ Orange CMTMRos (Invitrogen™) were used for fluorescence observation of the ER and mitochondria, respectively.

One possible factor is the difference in the location of PLP accumulated in cells. The lifetime of ^1^O_2_ produced in cells by the PDT process is short; they readily oxidize surrounding substances or are transformed into other ROS^2,14,22,24^. Therefore, the organelles where PLPs accumulate are rapidly affected. In particular, the generation of ROS near the organelles, such as cell nuclei and mitochondria, causes severe damage to biological activities. Therefore, to clarify the location of the dosed PLPs, we carried out fluorescence measurements using a confocal super-resolution microscope. Figure 4 shows the results, where a and f are endoplasmic reticulum (ER) fluorescence images, b and g are mitochondrial fluorescence images, c and h are PLP fluorescence images, d and i are merged mitochondrial and PLP images, and e and j are enlarged images of the squares in d and i for the RGK1 and RGM1 samples, respectively.

In the case of RGK1, the PLP fluorescence image contains bright spots and ring-shaped structures (inset of Fig. 4c)^17^, which are localized near mitochondria close to the nucleus, as shown in Fig. 4d, e. Because previous studies have shown that phagosomes are generated around the ER and mitochondria^25^, and that PLPs accumulate in phagosomes for RGK1^17^, the bright spots and ring-shaped structures observed here are considered to be phagosomes. The ring shape correspond to the cross section of the spherical structure of phagosomes obtained by confocal super-resolution microscope measurement, as shown in Fig. 1d^18^. In contrast, in the case of RGM1, although similar bright spots and ring-shaped structures, which are considered to be phagosomes, were formed, as shown in the inset of Fig. 4h, most of them are spread out in a band shape at the edge of the cell and do not overlap with the distribution of mitochondria, as shown in Fig. 4i, j.

Since PLPs are expected to distribute uniformly within cells when added, the observed difference in the distribution is considered to have occurred during the 24 h while the samples were allowed to stand in the incubator. Namely, some mechanism of autophagy may have caused the difference in the PLP-accumulated phagosome distribution. Therefore, we conducted experiments to further clarify the observed processes.

### PLP-induced phagosome generation and PLP capture processes

**Fig. 5:**
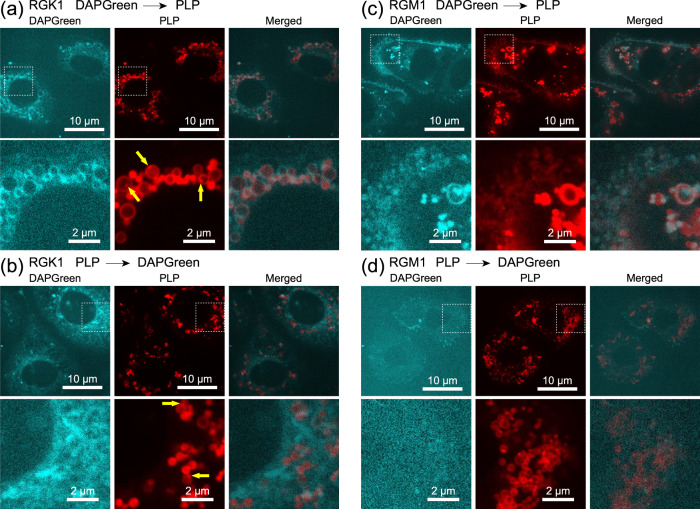
Double staining experiment with the autophagy detection reagent DAPGreen and PLP. **a** Fluorescence images of RGK1 administered with DAPGreen first and then PLP, **b** fluorescence images of RGK1 administered with PLP and then DAPGreen, **c** fluorescence images of RGM1 administered with DAPGreen and then PLP, and **d** fluorescence images of RGM1 administered with PLP and then DAPGreen. Yellow arrows indicate lysosomes. DAPGreen is a fluorescent probe that increases its fluorescence in response to a lipid-soluble environment. When DAPGreen is incorporated into the phagosome membrane, the lipid environment may change, leading to an increase in fluorescence. Indeed, in Fig. 5a, c, the phagosomes are stained with DAPGreen. On the other hand, in Fig. 5b, d, since the phagosomes do not take up PLP, the luminescence of DAPGreen does not match that of the phagosomes induced by PLP, and it is dispersed in a cell. In the case of Fig. 5b, d, as observed in the magnified images, a high-contrast, island-like structure was observed in RGK1, suggesting the effects of differences in the intracellular lipid-soluble environment. Phagosomes use the endoplasmic reticulum membrane as a material; therefore, they might be structurally related to the endoplasmic reticulum. Details exceed the scope of this paper, so we leave it as a topic for future research and will not delve further into it here.

First, to determine the formation processes of PLP-accumulated phagosomes, measurements with the autophagy detection reagent DAPGreen, which emits fluorescence when incorporated in the autophagosome membrane, and PLP fluorescence were performed. Figure 5 shows the results, where a comprises fluorescence images of RGK1 administered with DAPGreen first and then PLP, b comprises fluorescence images of RGK1 administered with PLP and then DAPGreen, c comprises fluorescence images of RGM1 administered with DAPGreen and then PLP, and d comprises fluorescence images of RGM1 administered with PLP and then DAPGreen.

For RGK1, when DAPGreen was administered first, the locations of DAPGreen and PLP fluorescence in the images were almost the same, as shown in Fig. 5a. They were observed as rings of various sizes with diameters of 0.3–2 μm, similarly to in Fig. 4. For RGM1, as similarly shown in Fig. 5c, the locations of DAPGreen and PLP fluorescencewere almost matched. However, unlike the case of RGK1, they were observed as ring structures with almost the same diameter of about 0.3 μm.

In contrast, when PLP was administered first, as shown in Fig. 5b, d, the locations of DAPGreen and PLP fluorescence did not match. In addition, the ring-shaped structures were observed in the PLP fluorescence measurement but not in the DAPGreen fluorescence measurement. These results indicate that the generation of phagosomes is triggered by PLP. In addition, in the case of RGK1, similarly to in previous results^17^, ring-shaped phagosomes are combined with lysosomes (indicated by yellow arrows in the figure) to form phagolysosomes, as shown in Fig. 5. On the other hand, for RGM1, only the ring-shaped phagosomes were observed, and no combination with lysosomes occurred.

These results are summarized as follows:

1. PLP initiates phagosome generation.
2. For both RGK1 and RGM1, PLP is incorporated in phagosome membranes.
3. The phagosomes in the RGK1 sample are large (0.3–2 μm) and located close to the cell nucleus and mitochondria, whereas the phagosomes in the RGM1 sample are small (0.3 μm), uniform in size, and located at the edge of the cell and spread out in a band shape.
4. Phagolysosomes are formed in RGK1 but not in RGM1.

**Fig. 6:**
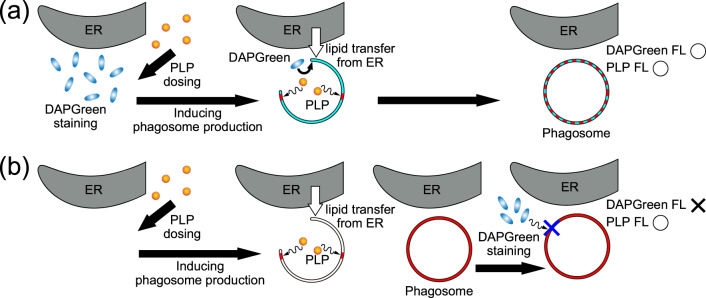
Model to explain the uptake of DAPGreen and PLP into the phagosome membrane. **a** DAPGreen administered first and **b** PLP administered first.

Regarding findings (1) and (2), the model shown in Fig. 6 is considered to explain the PLP-induced phagosome generation and PLP uptake. As shown in Fig. 6a, if DAPGreen is administered first, it remains unchanged in the cells because it does not induce phagosome generation. Then, when PLPs are administered subsequently, phagosome generation is induced. Phospholipids are supplied from the ER to phagosome precursors^25–28^, and DAPGreen is incorporated in the phagosome membrane together with PLPs^29^. Therefore, phagosome membranes are stained with DAPGreen and PLP after the phagosomes are formed. Namely, the locations of DAPGreen and PLP fluorescence are almost the same.

On the other hand, as shown in Fig. 6b, when PLP is administered first, phagosome generation is induced, and phospholipids are supplied from the ER to the phagosome precursors. Phagosomes take up PLP; thus, the phagosome membrane is stained with PLP after the phagosome are formed. Therefore, even after subsequent DAPGreen administration, the completely formed phagosomes do not incorporate DAPGreen. As a result, the phagosomes do not fluoresce with DAPGreen but with PLP, and, therefore, the locations of DAPGreen and PLP fluorescence do not match, as shown in Fig. 5b, d. PLP was found to be the factor that induces phagosome formation and to selectively accumulate in the phagosome membranes. As a result, the ring-shaped phagosome structure was observed in the fine z-stack structure shown in Fig. 1d. Time-lapse images obtained by a confocal super-resolution microscope using PLP fluorescence were later used to observe the light-induced dynamics of PLP-accumulated phagosomes in PDT.

### Size and distribution of PLP-accumulated phagosomes

Regarding the size difference indicated in finding (3), RGK1 has heterogeneous phagosome sizes, with many phagosomes exceeding 1 μm in diameter, whereas RGM1 has more uniform sizes with diameters around 0.3 μm. Although details have not yet been clarified, the phagosome size is determined by the expression level of ERdj8, a protein of the ER membrane^30^. Therefore, the expression levels of ERdj8 in RGK1 and RGM1 were compared by immunofluorescent staining.

**Fig. 7:**
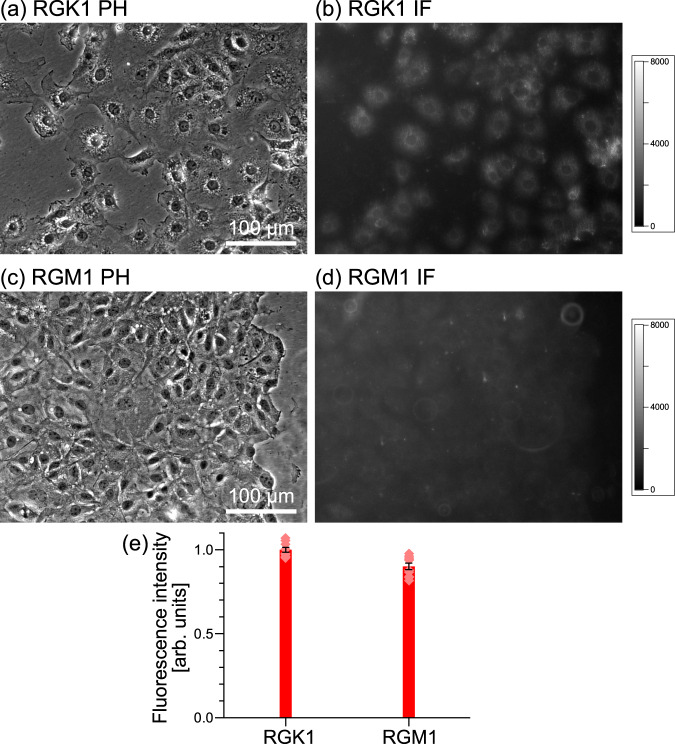
Analysis of the expression level of ERdj8, a protein of the ER membrane. **a**, **c** Phase contrast (PH) and **b**, **d** immunofluorescent (IF) staining images of RGK1 and RGM1, respectively. **e** Average fluorescence intensities of ten cells in **b** and **d**. The average fluorescence intensity of RGK1 is 11 % higher than that of RGM1. From this comparison, it can be inferred that RGK1 has a higher expression level of ERdj8 than RGM1, supporting the observed difference in the phagosome size.

Figure 7a, c shows phase-contrast images of RGK1 and RGM1, and Fig. 7b, d shows immunofluorescence staining images of RGK1 and RGM1, respectively. Figure 7e represents the average fluorescence intensities of ten cells in Fig. 7b, d. The average fluorescence intensity of RGK1 is 11 % higher than that of RGM1. From this comparison, it can be inferred that RGK1 has a higher expression level of ERdj8 than RGM1, supporting the observed difference in the phagosome size.

Regarding the distribution difference indicated in finding (3), as was pointed out in the previous section, since PLP is expected to distribute uniformly within cells when added, the observed difference in the distribution is considered to occur during the 24 h while the samples were allowed to stand in the incubator. Namely, the PLP-accumulated phagosomes may have moved toward the center of cells in RGK1 and toward the edge of the cells in RGM1.

In autophagy, the state of cellular starvation affects the process^31–35^. Motor proteins drive phagosomes to travel along microtubules, the main intracellular transportation routes within a cell^36–43^. When a cell is in a state of starvation, a phagosome merges with a lysosome to form a phagolysosome^18,44–48^. The lysosome is a digestive organ of the cell, breaking down and reusing molecules such as parts of organelles, proteins, lipids, and sugars taken up by the phagosome.

Moreover, the phagosome membrane contains LC3 (microtubule-associated protein light chain 3)^40,49,50^, which binds to microtubules through the scaffolding protein JIP1 (JNK-interacting protein 1)^40^. JIP1 was originally identified for its ability to recruit multiple kinases in the JNK pathway, it had been suggested that JIP1 regulates constitutive axonal transport^36,39–43,51^. If the cell is a state of starvation, phagosome tends to move toward the centrosome along the microtubules^31,32,34,35^. If the cell is not in a state of starvation, phagosome tends to move toward the edge of the cell along the microtubules^31,32,34–38,40,41,52^. This mechanism is consistent with the observation that phagosomes accumulate at the center in RGK1 but localize at the edge of the cell in RGM1.

Furthermore, it is known that STX17 (Syntaxin 17), a membrane protein, plays a crucial role in the formation of phagolysosomes^33,48,53^. In a state of starvation, STX17 reduces its localization to mitochondria and ER and accumulates on the phagosome membrane;^33,53^ thus, the phagosome binds to the lysosome via STX17^48^. On the other hand, when the cell has sufficient energy, little STX17 accumulates on the phagosome membrane, making it difficult for the phagosome to bind to the lysosome. This mechanism aligns well with the observation of phagolysosomes in RGK1, and the phagosomes remain in RGM1.

**Fig. 8:**
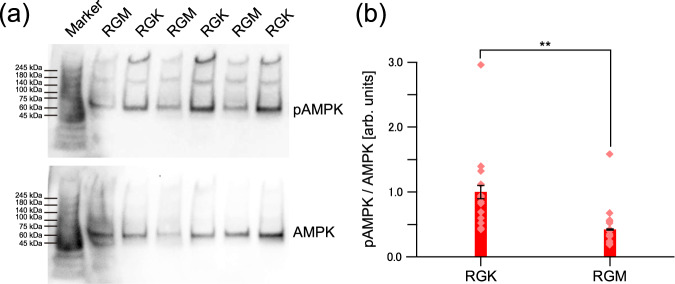
Western blot analysis of AMPK-activated protein kinase α (AMPK) and its phosphorylated (activated) form α (pAMPK) for RGK1 and RGM1. **a** Representative western blot images. Western blot images including protein molecular weight markers are shown in Supplementary Fig. S1. **b** Ratio of pAMPK to AMPK estimated from the western blot images. **p < 0.01.

From the above considerations, if cancer cells are in a state of starvation, the formation and distribution of phagosomes and phagolysosomes in RGK1 and RGM1 can be well explained. To determine the starvation status of RGK1 and RGM1, we examined the activity of AMPK (adenosine monophosphate-activated protein kinase) in the cells. It is known that when cells are in a state of starvation, such as when there is insufficient ATP, AMPK is phosphorylated (activated) to become pAMPK^54^. Therefore, we performed western blot measurements of pAMPK/AMPK in RGK1 and RGM1. Figure 8 comprises (a) a representative western blot image and (b) the ratio of pAMPK to AMPK estimated from the western blot. As shown in Fig. 8b, the activity of pAMPK is high in RGK1. That is, the result that phagolysosomes are formed only in RGK1 aligns well with the interpretation that the RGK1cancer cells are in a state of starvation.

### Light-driven dynamics of PLP-accumulated phagosomes in PDT

**Fig. 9:**
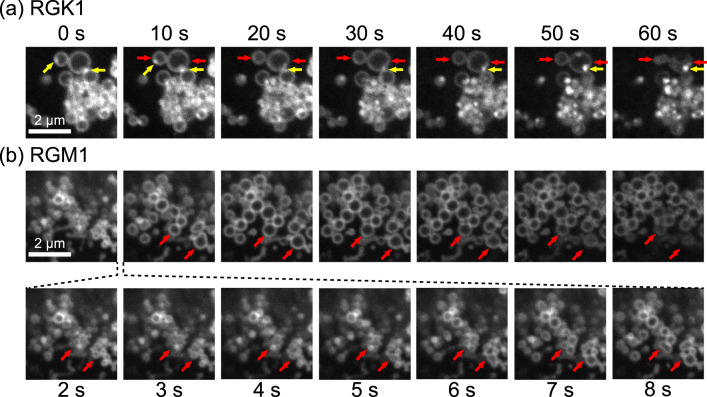
Time-lapse images of phagosomes with PDT. **a** RGK1 and **b** RGM1 phagosomes during 1 min light irradiation (337 mW cm^-2^). The frame rate is 2 fps. In RGK1, the phagolysosome, which was formed by the fusion of the phagosome indicated by the red arrow and the lysosome indicated by the yellow arrow, maintained its spherical shape for up to 50 s, then was destroyed. In RGM1, small phagosomes fused with the surrounding phagosomes to become larger ones with a diameter of 0.5–1 μm during the first 10 s. After 10 s, the phagosomes maintained their spherical shape and size and did not break; thus, they did not damage the cells. Supplementary Movie S1 in the Supplementary shows this time variation.

Finally, to examine in detail the effect of PDT observed at the cellular level shown in Fig. 2 at the level of the phagosome, we observed the dynamics of phagosomes during 1 min light irradiation. Figure 9a, b shows the time-lapse images obtained for RGK1 and RGM1, respectively. As shown in Fig. 9a, phagolysosomes, namely, phagosomes bound to lysosomes indicated by yellow arrows, maintained their spherical shape while deforming up to 50 s, after which they were destroyed. In contrast, as shown in Fig. 9b, small phagosomes fused with the surrounding phagosomes during the first 10 s bind to become larger ones with a diameter of 0.5–1 μm. They were preserved and not destroyed.

Here, upon light irradiation, singlet oxygen was generated by the chlorin ring of pyropheophorbide-lipid accumulated in the phagosome membrane, and the phagosome membrane was oxidized and deformed. After 50 s, the cage structure of the phagolysosome could no longer be maintained and was destroyed. Thereby, the internal hydrolases and ROS leaked out, damaging the cell^17^.

In contrast, in the case of RGM1 phagosomes shown in Fig. 9b, the administered PLP induced the generation of small phagosomes as indicated by the low expression level of ERdj8^30^. Phagosome precursors surrounded the PLP and concomitantly accumulated the PLP in the phagosome membrane. When PDT was performed and light was irradiated, the surrounding phagosomes fused and transformed into larger phagosomes within the first 10 s. Since the lysosomes did not bind to phagosomes, ROS and hydrolytic enzymes did not leak into the cell during fusion. After 10 s, the phagosomes maintained their spherical shape and size and did not break; thus, they did not damage the cells.

The size of the completed phagosome depends on the expression level of ERdj8, it is dependent on the normal state in which the cells are not exposed to oxidative stress. Therefore, it is suggested that the observed change in the size of phagosomes is considered to be subjected to oxidative stress. As shown in Fig. 9b, the phagosomes did not grow indefinitely during the light irradiation, and their growth stopped after about 20 s. This cessation of growth may be due to physical or structural limitations, such as phospholipid packing due to oxidation of the phagosome membrane.

These factors are considered to be responsible for the observed differences in the effects of PLP-PDT on RGK1 and RGM1.

## Discussion

**Fig. 10:**
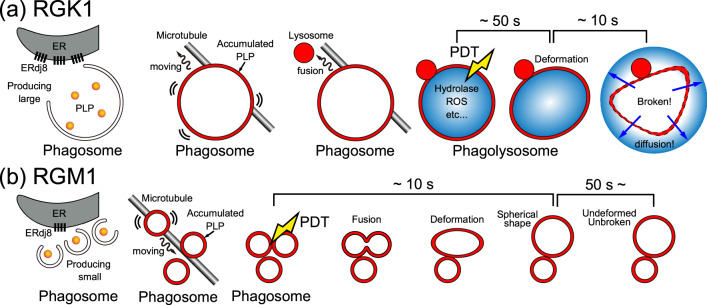
Schematic diagrams to explain the phagosome generation and reaction processes in PDT. **a** RGK1 and **b** RGM1. In both cases, administered PLPs induce phagosome generation, where PLPs accumulate on the phagosomal membrane. The phagosome size depends on the level of ERdj8 expression, as shown in Fig. 7, with larger phagosomes (0.3–2 μm) in RGK1 and smaller phagosomes (~0.3 μm) formed in RGM1. The phagosomes move along the microtubules. In RGK1, under starvation conditions, they move towards the cell center, merging with lysosomes to form phagolysosomes, leading to the digestion of their contents. In RGM1, they move toward the periphery of the cell and do not merge with lysosomes. When PDT is performed under such conditions, in RGK1, the membrane of the phagosome ruptures and its contents are released. The released contents considerably damage the cell, leading to necrosis, as shown in Fig. 2. In contrast, in RGM1, smaller phagosomes merge to form larger ones. During the experiment, the phagosomes were not destroyed. These processes are considered to be responsible for the difference in PDT observed in Fig. 2.

On the basis of the obtained results, the dynamics shown in Fig. 10 is considered to explain the PDT effect when using PLP. In both cases, administered PLP induces phagosome generation, where PLP accumulates on the phagosome membrane. The phagosome size depends on the level of ERdj8 expression, with larger phagosomes (0.3 ~ 2 μm) formed in RGK1 and smaller phagosomes (~0.3 μm) in RGM1.

As explained in the section titled “Size and distribution of PLP-accumulated phagosomes” in the main text: (1) Phagosomes induced by PLP are widely distributed within the cell upon formation. Since RGK1 is a cancer cell and in the starved state, they move towards the near the cell nucleus, while in RGM1 is a normal cell and not in the starved state, they move towards the periphery. (2) Lysosomes are formed by the Golgi apparatus and hence are mainly found near the nucleus, though some may be located at the periphery. (3) Phagosomes only bind with lysosomes once STX17 localizes on their membrane. (4) STX17 exists at the mitochondria and the endoplasmic reticulum and is localizes on phagosomes in the starved state (RGK1). (5) Therefore, it is likely that RGM1 is not in a starved state as confirmed in Fig. 8 and phagosomes rarely bind with lysosomes. (6) Lysosomes only incorporate and emit light from PLP after binding with phagosomes. Thus, the probability that the bright spots in RGM1 are lysosomes is low. Also, in RGM1, smaller phagosomes combine to grow in size. This result suggests overlapping regions between adjacent phagosomes can be seen as bright spots. Smaller phagosomes can be seen as bright spots, too. Similarly, in RGK1, small phagosomes or overlapping regions between adjacent phagosomes might also be visible as bright spots.

The brightness of the emission depends on the integrated value of PLP-induced luminescence in the z-direction. The super-resolution confocal microscope used in our experiments has a high resolution in the z-direction, which means only the focused layer is visible. Phagosomes appear ring-shaped due to their spherical membrane structure, and their shape changes when the focal point shifts due to factors like drift. While phagosomes and lysosomes merge by binding their membranes, the lysosome portion appears as a small clump first. This process may lead to a larger integrated value in the z-direction, making it seem brighter. Smaller phagosomes similarly appear as bright spots.

When PDT is performed under such conditions, in RGK1, the membrane of the phagosome ruptures and its contents are released. The released contents considerably damage the cell, leading to the necrosis shown in Fig. 2. In contrast, in RGM1, smaller phagosomes merge to form larger ones. During the experiment, the phagosomes were not destroyed. For PDT using PLP, a new mechanism utilizing the autophagy pathway was discovered. This finding provides a new understanding of various aspects of the treatment, including the highly selective and potential effects of PLP-PDT.

Autophagy, which serves to normalize cellular functions by removing misfolded proteins, among other substances, has a tumor-suppressing effect in early-stage cancer^55–58^. Therefore, inducing the formation of phagosomes can lead to tumor suppression. On the other hand, in growing tumors, autophagy acts as a crucial survival pathway, providing nutrients and eliminating anti-cancer agents^55–57,59^. Therefore, controlling the autophagy in tumors can be an effective treatment^55–59^. Moreover, the combination of PLP and PDT, which selectively controls phagosomes, can potentially contribute to the treatment of various diseases associated with autophagy.

In conclusion, we have revealed that PLP enables highly effective cancer treatment through PDT by employing a previously unreported PDT mechanism that utilizes the process of autophagy. We revealed that PLP enables highly effective cancer treatment through PDT by employing a unique mechanism that utilizes the process of autophagy. The dynamics of PLP-accumulated phagosomes immediately after light irradiation were found to be completely different between normal cells and cancer cells, and it became clear that this difference results in the manifestation of the characteristic effect of PDT when using PLP.

Since PLP exhibits luminescence of sufficient intensity even after its accumulation on a phagosome membrane, PLP is expected to play an important role in therapies targeting autophagy and as a highly efficient photosensitizer for PDT. PLP was originally developed as a drug delivery agent. This study revealed the potential for intracellular drug delivery processes through PLP-induced autophagy. This discovery could potentially pave the way for entirely new possibilities in the future. Other biological materials might also possess this mechanism. However, to exhibit effects like PLP, it is considered that the following conditions must be met: (1) They must have a structure that allows efficient uptake by cells. (2) They must be substances that induce autophagy. (3) They must be materials that accumulate on the phagosome membrane. Further research into these aspects is desirable.

## Methods

### Preparation of polphylipoprotein (PLP)

PLP is a photosensitizer that contains a hydrophobic drug-loadable core enveloped in a porphyrin–lipid monolayer and constrained by ApoA-1 mimetic R4F (Ac-FAEKFKEAVKDYFAKFWD) peptide networks^16,17,20^.

### Culturing cells

RGM1, a rat gastric epithelial cell line, was purchased from RIKEN CELLBANK (Ibaraki, Japan). The RGK1 cells used in this study were rat gastric mucosa-derived cancer-like mutant cells^21^, a chemically induced oncogenic cancer-like mutant of RGM1^21^. RGK1 cells were cultured in DMEM/F12 without L-glutamine (Sigma-Aldrich Japan K.K., Tokyo, Japan). RGM1 cells were cultured in DMEM/F12 with L-glutamine (Gibco^TM^, Thermo Fisher Scientific K.K.).

### Sample preparation

PLP (final concentration 19 μM) was added to RGK1 and RGM1 cells cultured in a plastic dish of 60 mm diameter (for phase-contrast observation) or a glass bottom dish of 35 mm diameter (for super-resolution observation) and allowed to stand for 24 h in an incubator at 37 °C with a CO_2_ concentration of 5%. This concentration of PLP is not toxic^20^. In a previous experiment in which the incubation time was varied^20^, it was confirmed that PLP dose does not saturate in 3 h but saturates in 12 h. In this study, we performed incubation for 24 h so that the amount of sensitizer entering the cells was saturate. For talaporfin sodium, for example, the quantum yield is the same in both normal and cancer cells, and the difference in uptake is being utilized^14^. In this study, we adjusted the amount of PLP taken up by RGK1 and RGM1 to be equivalent. When PLP is incorporated into the phagosomal membrane, it is degraded. As shown in Fig. 3–5, there is no significant difference in luminescence intensity when using PLP in RGK1 and RGM1. This result suggests that the quantum yield of PLP is almost equivalent. The experiment’s validity, where the uptake amount of PLP was adjusted for RGK1 and RGM1 to make the concentrations of both equivalent, is supported.

### Measurement of PLP concentration in a cell

RGK1 and RGM1 were cultured in a 24-well-plate, then PLP (final concentration 19 μM) was added, followed by incubation for 24 h. After each well was washed twice with PBS, 100 μL of RIPA buffer was added and the cells in the well were lysed. The cell lysate was moved to a 96-well plate, and the fluorescence intensity was measured by a microplate reader. The excitation and fluorescence wavelengths were 420 and 675 nm, respectively. Since the number of cells cultured in each well differed, the fluorescence intensity was normalized by the number of cells in each well. Since the amount of total protein in each well is related to the number of cells, the amount of total protein in each well after fluorescence measurement was analyzed using the BCA Protein Assay Kit (TaKaRa Bio Inc.) to normalize the PLP data.

### Endoplasmic reticulum and mitochondria staining

ER-Tracker™ Green (Invitrogen™) and MitoTracker™ Orange CMTMRos (Invitrogen™) were used for fluorescence staining of the ER and mitochondria, respectively. PLP-added RGK1 and RGM1 cells were cultured in a glass-bottom dish of 35 mm diameter and allowed to stand for 24 h in an incubator at 37 °C with a CO_2_ concentration of 5%. Next, 0.5 μL of ER Tracker DMSO solution at 1 mM concentration and 0.5 μL of MitoTracker DMSO solution at 1 mM concentration were added to 2 mL of Hank’s balanced salt solution (Gibco^TM^, Thermo Fisher Scientific K.K.). Then this solution was replaced with the medium in the dish and allowed to stand for 10 min in an incubator. Finally, the staining solution in the dish was replaced with the medium, and the sample was observed using a super-resolution microscope.

### DAPGreen staining

DAPGreen (Dojindo Laboratories Co., Ltd.) detects autophagosomes and autolysosomes. DAPGreen is incorporated in autophagosomes during double membrane formation and emit fluorescence under hydrophobic conditions.

The cells cultured in a glass-bottom dish shown in Fig. 5a, b were allowed to stand in an incubator for 24 h after PLP dosing. After washing the sample twice with a new medium without PLP, 2 mL of the medium was added and 2 μL of 0.1 mM DAPGreen DMSO solution was added. After being allowed to stand in the incubator for 30 min, the medium in the dish was removed by washing twice with a new medium without DAPGreen. Then, 2 mL of the medium was added, and the sample was observed using a super-resolution microscope.

For the cells in Fig. 5b, d, the DAPGreen staining and PLP dosing were reversed. Two milliliters of the medium with 2 μL of 0.1 mM DAPGreen DMSO solution was replaced with the medium in the dish. After being allowed to stand in the incubator for 30 min, the medium in the dish was removed, washed twice with the　new medium, and 2 mL of the medium with PLP was added. After being allowed to stand in an incubator for 3 h, the medium in the dish was removed, washed twice with new medium, the sample was observed using a super-resolution microscope.

### PDT and time-lapse observations

Phase-contrast and time-lapse observations were performed with an IX83 microscope system (Olympus Corp.) equipped with a stage-top incubator (Tokai Hit Co., Ltd.). A super-resolution observation unit (Yokogawa Electric Corp., CSU-W1 SoRa) was installed in the IX83 system and used to observe sites where PLP accumulated. For the light irradiation of PDT, we used the white light source of the IX83 system for fluorescence observation, with a 655 nm filter and a 640 nm semiconductor laser in the CSU-W1 SoRa system.

### Western blotting analysis

RGK1 and RGM1 samples were placed in a culture dish with a diameter of 60 mm and allowed to stand in an incubator for two days. Proteins were extracted from these samples using RIPA buffer, and western blotting was performed. The primary antibody phospho-AMPKα (Thr172) (40H9) rabbit mAb (Cell Signaling Technology) and AMPKα (D5A2) Rabbit mAb (Cell Signaling Technology) were used for the detection of pAMPK and total AMPK, respectively.

### Reporting summary

Further information on research design is available in the Nature Portfolio Reporting Summary linked to this article.

### Supplementary information

Supplementary information

Description of Additional Supplementary Files

Supplementary Data 1

Supplementary Data 2

Supplementary Data 3

Supplementary Movie 1

Reporting Summary

## Data Availability

Numerical source data for Fig. 3, Fig. 7, and Fig. 8 can be found in Supplementary Data 1, Supplementary Data 2 and Supplementary Data 3, respectively.
