# Supplementary material for: Polphylipoprotein-induced autophagy mechanism with high performance in photodynamic therapy
Source: Commun Biol. 2023 Nov 28;6:1212. doi: 10.1038/s42003-023-05598-0 (PMC10684771; doi:10.1038/s42003-023-05598-0)
Supplement: Supplementary file 1 — Supplementary information [file 42003_2023_5598_MOESM1_ESM.pdf]

Supplementary information

Polphylipoprotein-induced autophagy mechanism with high performance in photodynamic therapy

Atsushi Taninaka[1,2], Hiromi Kurokawa[3], Mayuka Kamiyanagi[1], Takahiro Ochiai[2], Yusuke Arashida[1], Osamu Takeuchi[1], Hirofumi Matsui[3], and Hidemi Shigekawa[1,*]

1 Faculty of Pure and Applied Sciences, University of Tsukuba, 1-1-1 Tennodai, Tsukuba, Ibaraki, 305-8573 Japan

2 TAKANO Co. LTD. Miyada-mura, Kamiina-gun, Nagano, 399-4301 Japan

3 Fuculty of Medicine, University of Tsukuba, 1-1-1 Tennodai, Tsukuba, Ibaraki, 305-8575 Japan

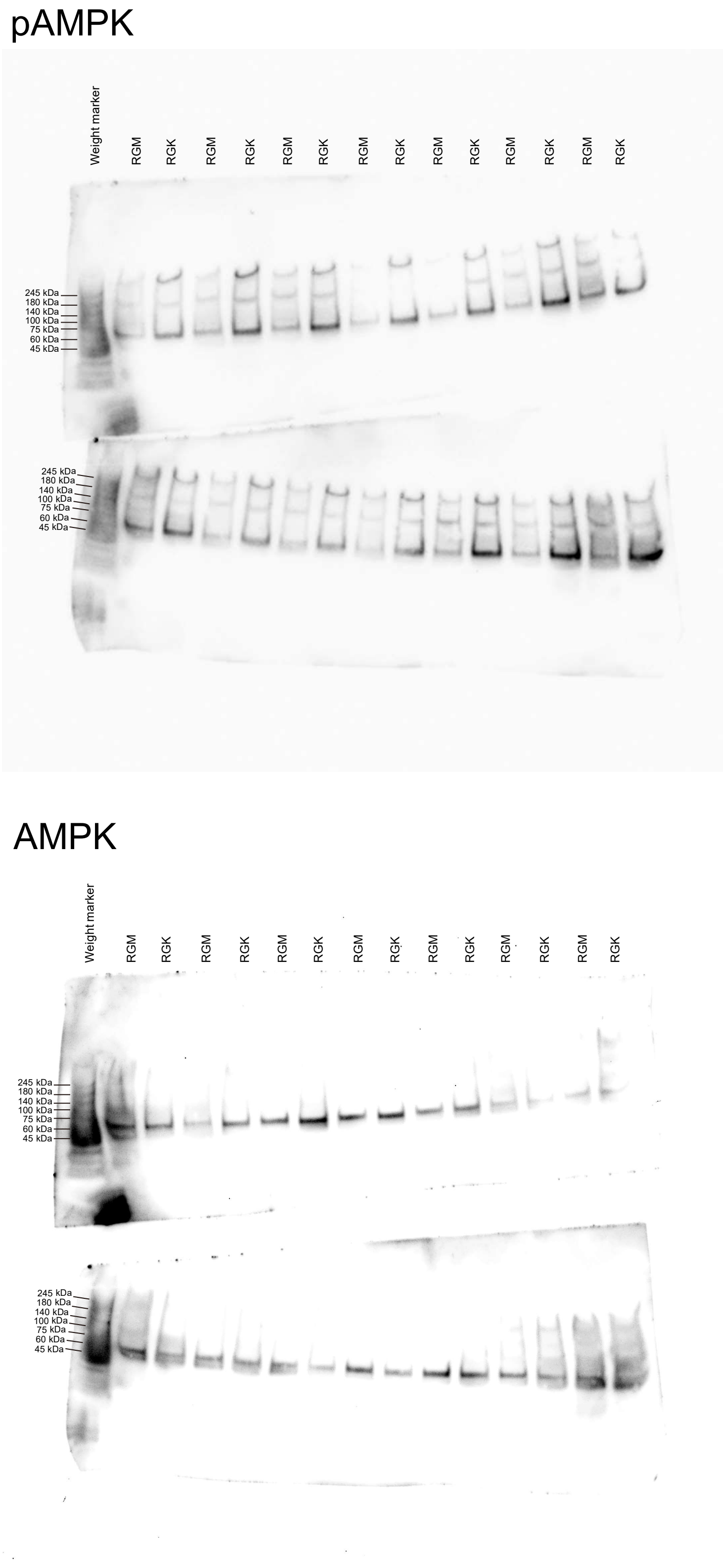

Figure S1. Western blot images including protein molecular weight markers.

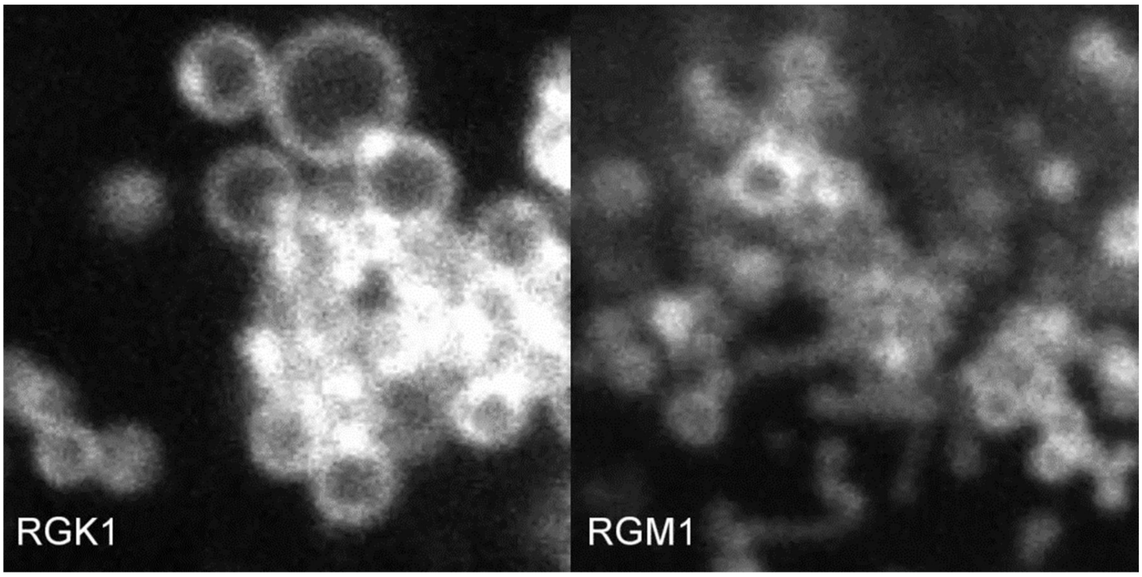

Movie S1. Time-lapse movie of RGK1 and RGM1 phagosomes during 1 min light irradiation (337 mW cm^{-2}). The frame rate is 2 fps. The super-resolution confocal microscope used for observation has a high resolution in the Z-direction, and fluorescence not within the focal depth is not observed as a bright spot. As a result, the image changes due to thermal drift between the objective lens and the sample during the measurement or due to the movement of the phagosome. For example, from around 50 s to 55 s, a white dot appears at the top end of the phagosome, indicated by the red arrow on the right side of the RGK1 image. It is considered that lysosomes, which were not within the focal depth at the start of the time-lapse, were observed because they entered the focal range. This phagosome is large and may have two attached lysosomes. In that case, they would have a similar effect as a phagolysosome, and both are related to the degradation. At 60 s, the lysosome in the bottom right appears as the remnants of a degraded phagolysosome membrane. The focal point of this experiment is the formation of phagosomes induced by PLP. PLP is incorporated into and degraded on the membrane of the phagosomes formed by its introduction. Once a phagosome binds with a lysosome, the membranes of both entities merge to make a phagolysosome. The degraded PLP is introduced into the membrane areas of the lysosome, making the entire structure emit light. Hence, experiments to demonstrate the co-localization of phagosomes and lysosomes are not straightforward compared to the case shown in Fig. 4, where two materials exist separately. Therefore, such analysis is left to a subject for future studies.
