# Supplementary material for: Polphylipoprotein-induced autophagy mechanism with high performance in photodynamic therapy
Source: Commun Biol. 2023 Nov 28;6:1212. doi: 10.1038/s42003-023-05598-0 (PMC10684771; doi:10.1038/s42003-023-05598-0)
Supplement: Supplementary file 2 — Description of Additional Supplementary Files [file 42003_2023_5598_MOESM2_ESM.pdf]

Description of Additional Supplementary Files

File name: Supplementary Data 1

Description: Ratio of PLP taken into cells to the dose for RGK1 and RGM1 determined by the fluorescence intensity of PLP.

File name: Supplementary Data 2

Description: Fluorescence intensities obtained for RGK1 and RGM 1

File name: Supplementary Data 3

Description: Ratios of pAMPK to AMPK estimated from the western blot images for RGK1 and RGM 1.

File name: Supplementary Movie 1

Description: Time-lapse movie of RGK1 and RGM1 phagosomes during 1 min light irradiation (337 mW cm-2).
