# Supplementary material for: Polphylipoprotein-induced autophagy mechanism with high performance in photodynamic therapy
Source: Commun Biol. 2023 Nov 28;6:1212. doi: 10.1038/s42003-023-05598-0 (PMC10684771; doi:10.1038/s42003-023-05598-0)
Supplement: Supplementary file 7 — Reporting Summary [file 42003_2023_5598_MOESM7_ESM.pdf]

nature portfolio

Corresponding author(s): Hidemi Shigekawa
Last updated by author(s): Nov 4, 2023

# Reporting Summary

Nature Portfolio wishes to improve the reproducibility of the work that we publish. This form provides structure for consistency and transparency in reporting. For further information on Nature Portfolio policies, see our Editorial Policies and the Editorial Policy Checklist.

## Statistics

For all statistical analyses, confirm that the following items are present in the figure legend, table legend, main text, or Methods section.

| n/a | Confirmed | |
|---|---|---|
| ☐ | ☒ | The exact sample size (*n*) for each experimental group/condition, given as a discrete number and unit of measurement |
| ☐ | ☒ | A statement on whether measurements were taken from distinct samples or whether the same sample was measured repeatedly |
| ☐ | ☒ | The statistical test(s) used AND whether they are one- or two-sided<br>*Only common tests should be described solely by name; describe more complex techniques in the Methods section.* |
| ☒ | ☐ | A description of all covariates tested |
| ☒ | ☐ | A description of any assumptions or corrections, such as tests of normality and adjustment for multiple comparisons |
| ☐ | ☒ | A full description of the statistical parameters including central tendency (e.g. means) or other basic estimates (e.g. regression coefficient) AND variation (e.g. standard deviation) or associated estimates of uncertainty (e.g. confidence intervals) |
| ☐ | ☒ | For null hypothesis testing, the test statistic (e.g. *F*, *t*, *r*) with confidence intervals, effect sizes, degrees of freedom and *P* value noted<br>*Give P values as exact values whenever suitable.* |
| ☒ | ☐ | For Bayesian analysis, information on the choice of priors and Markov chain Monte Carlo settings |
| ☒ | ☐ | For hierarchical and complex designs, identification of the appropriate level for tests and full reporting of outcomes |
| ☒ | ☐ | Estimates of effect sizes (e.g. Cohen's *d*, Pearson's *r*), indicating how they were calculated |

*Our web collection on statistics for biologists contains articles on many of the points above.*

## Software and code

Policy information about availability of computer code

| | |
|---|---|
| Data collection | MetaMorph imaging software<br>Gen 5 Microplate Reader and Imager Software 3.09<br>Vilber Lourmat (FUSION FX7.EDGE) |
| Data analysis | Image J 1.53t<br>Igor Pro 6.36 |

For manuscripts utilizing custom algorithms or software that are central to the research but not yet described in published literature, software must be made available to editors and reviewers. We strongly encourage code deposition in a community repository (e.g. GitHub). See the Nature Portfolio guidelines for submitting code & software for further information.

## Data

Policy information about availability of data

All manuscripts must include a data availability statement. This statement should provide the following information, where applicable:

- Accession codes, unique identifiers, or web links for publicly available datasets
- A description of any restrictions on data availability
- For clinical datasets or third party data, please ensure that the statement adheres to our policy

All data supporting the findigs of this study are included in the article and its supplementary information.

## Research involving human participants, their data, or biological material

Policy information about studies with human participants or human data. See also policy information about sex, gender (identity/presentation), and sexual orientation and race, ethnicity and racism.

| | |
|---|---|
| Reporting on sex and gender | No applicable |
| Reporting on race, ethnicity, or other socially relevant groupings | No applicable |
| Population characteristics | No applicable |
| Recruitment | No applicable |
| Ethics oversight | No applicable |

Note that full information on the approval of the study protocol must also be provided in the manuscript.

# Field-specific reporting

Please select the one below that is the best fit for your research. If you are not sure, read the appropriate sections before making your selection.

☒ Life sciences ☐ Behavioural & social sciences ☐ Ecological, evolutionary & environmental sciences

For a reference copy of the document with all sections, see nature.com/documents/nr-reporting-summary-flat.pdf

# Life sciences study design

All studies must disclose on these points even when the disclosure is negative.

| | |
|---|---|
| Sample size | Sample size was determined according to widely used protocols |
| Data exclusions | No data were excluded from the analyses |
| Replication | Each experiment was repeated at least three times |
| Randomization | No samples were randomized |
| Blinding | No samples were randomized |

# Reporting for specific materials, systems and methods

We require information from authors about some types of materials, experimental systems and methods used in many studies. Here, indicate whether each material, system or method listed is relevant to your study. If you are not sure if a list item applies to your research, read the appropriate section before selecting a response.

## Materials & experimental systems

| n/a | Involved in the study | |
|---|---|---|
| ☐ | ☒ | Antibodies |
| ☐ | ☒ | Eukaryotic cell lines |
| ☒ | ☐ | Palaeontology and archaeology |
| ☒ | ☐ | Animals and other organisms |
| ☒ | ☐ | Clinical data |
| ☒ | ☐ | Dual use research of concern |
| ☒ | ☐ | Plants |

## Methods

| n/a | Involved in the study | |
|---|---|---|
| ☒ | ☐ | ChIP-seq |
| ☒ | ☐ | Flow cytometry |
| ☒ | ☐ | MRI-based neuroimaging |

## Antibodies

| | |
|---|---|
| Antibodies used | phospho-AMPKα (Thr172) (40H9) rabbit mAb (Cell Signaling Technology)<br>AMPKα (D5A2) Rabbit mAb (Cell Signaling Technology)<br>rabbit anti-DNAJC16 (ERdj8) (17599–1-AP) (Proteintech) |

| | |
|---|---|
| Validation | The following primary antibody validation information can be found in the manufacturer's website.<br>https://www.cellsignal.jp/products/primary-antibodies/phospho-ampka-thr172-40h9-rabbit-mab/2535<br>https://www.cellsignal.jp/products/primary-antibodies/ampka-d5a2-rabbit-mab/5831<br>https://www.ptglab.co.jp/products/DNAJC16-Antibody-17599-1-AP.htm |

## Eukaryotic cell lines

Policy information about cell lines and Sex and Gender in Research

| | |
|---|---|
| Cell line source(s) | RGM1, a rat gastric epithelial cell line, was purchased from RIKEN CELLBANK.<br>The RGK1 cells used in this study were rat gastric mucosa-derived cancer-like mutant cells, a chemically induced oncogenic cancer-like mutant of RGM1[ref. 21].<br><br>[ref. 21] Shimokawa, O. et al. In Vitro Cellular & Developmental Biology - Animal 44, 26–30 (2008). |
| Authentication | Cell line received directly from RIKEN CELLBANK |
| Mycoplasma contamination | Cell line tested for Mycoplasma contamination upon receiving cell line from RIKEN CELLBANK. |
| Commonly misidentified lines<br>(See ICLAC register) | No misidentified lines were used |
